# The Active Inference Approach to Ecological Perception: General Information Dynamics for Natural and Artificial Embodied Cognition

**DOI:** 10.3389/frobt.2018.00021

**Published:** 2018-03-08

**Authors:** Adam Linson, Andy Clark, Subramanian Ramamoorthy, Karl Friston

**Affiliations:** ^1^Department of Computing Science and Mathematics, University of Stirling, Stirling, United Kingdom; ^2^Department of Philosophy, University of Stirling, Stirling, United Kingdom; ^3^Institute for Advanced Studies in the Humanities, University of Edinburgh, Edinburgh, United Kingdom; ^4^School of Philosophy, Psychology and Language Sciences, University of Edinburgh, Edinburgh, United Kingdom; ^5^Department of Philosophy, Macquarie University, Sydney, NSW, Australia; ^6^School of Informatics, University of Edinburgh, Edinburgh, United Kingdom; ^7^Edinburgh Centre for Robotics, Edinburgh, United Kingdom; ^8^The Wellcome Trust Centre for Neuroimaging, University College London, London, United Kingdom

**Keywords:** free energy, uncertainty, self-organization, embodiment, evolution, affordances, skilled expertise, frame problem

## Abstract

The emerging neurocomputational vision of humans as embodied, ecologically embedded, social agents—who shape and are shaped by their environment—offers a golden opportunity to revisit and revise ideas about the physical and information-theoretic underpinnings of life, mind, and consciousness itself. In particular, the active inference framework (AIF) makes it possible to bridge connections from computational neuroscience and robotics/AI to ecological psychology and phenomenology, revealing common underpinnings and overcoming key limitations. AIF opposes the mechanistic to the reductive, while staying fully grounded in a naturalistic and information-theoretic foundation, using the principle of free energy minimization. The latter provides a theoretical basis for a unified treatment of particles, organisms, and interactive machines, spanning from the inorganic to organic, non-life to life, and natural to artificial agents. We provide a brief introduction to AIF, then explore its implications for evolutionary theory, ecological psychology, embodied phenomenology, and robotics/AI research. We conclude the paper by considering implications for machine consciousness.

## 1 Overview and Gentle Introduction to the Active Inference Framework (AIF)

In this article, we will consider the active inference framework (AIF)—or, more strictly speaking, the principle of free energy minimization (FEM)—as a principle, rather than as a hypothesis. This means that we do not consider evidence for or against AIF *per se*. As a principle, AIF cannot be falsified—it is just a formal description of dynamics (much like Hamilton’s principle of least action; see below) that we apply to sentient agents. The process theories that attend AIF do, clearly, require evidence, which we refer to in our discussion.

Following a general overview, this section offers a gentle introduction to AIF, illustrating aspects of its instantiation as predictive processing (PP). Subsequent sections unpack the framework in greater detail, drawing out its implications for evolutionary theory, ecological psychology, embodied phenomenology, and robotics/AI research. In the final section, we directly consider aspects of machine consciousness.

Given the ill-defined concept of consciousness, we endeavor to bring onto the same page researchers from physics, biology, neuroscience, philosophy, cognitive science, and robotics/AI, by reviewing concepts that are sometimes presumed to have unique and self-evident meanings. This approach aims to dispel misinterpretations and sharpen the cross-disciplinary focus on the substance of the claims. Throughout the following exposition and argument, there are several deep connections to the possibility of machine consciousness, although this topic only emerges as central in the concluding section. The preliminary sections are a necessary prelude to appreciating the implications of AIF for biology and robotics/AI, given that the notion of consciousness in robotics is sourced from the human equivalent. It is, therefore, important to establish a perspective from which human biology is accounted for by a mechanistically grounded, information-theoretic treatment. This perspective can lend itself to robotic implementation; however, without this grounding, any arbitrary properties associated with consciousness could be thusly implemented, putting the proverbial cart before the horse in modeling the target phenomenon.

Embodied and embedded human cognition has been analyzed extensively, not only in cognitive science but also in ecological psychology and phenomenological philosophy. Furthermore, all three fields have continually engaged with robotics/AI, contributing insights and critical perspectives, in some cases even effecting technological shifts (see, e.g., Brooks, 1999; Dreyfus, 2007; see also Chemero and Turvey, 2007; Sahin et al., 2007). More recently, there has been a proliferation of fruitful exchanges between robotics/AI and neuroscience (Hassabis et al., 2017), especially with respect to PP. The generalization of PP in AIF makes it possible to bridge connections to ecological psychology and phenomenology, revealing common underpinnings and overcoming key limitations inherent to the latter two.

To indicate where this account is headed, our conclusion supports the idea that there is a fundamental relationship between (self-)consciousness and processual recursion, which has been suggested in other work (e.g., Maturana, 1995; Seth et al., 2006). To reach this conclusion, our discussion of consciousness is deferred throughout the paper, which tries to account for the emergence of processes and recursive architectures that underwrite a conscious embodied agent. In this light, we set up AIF in Section 1 in such a way as to be expanded upon in later sections. Sections 2 and 3 take a long view of the emergence of human biology that paves the way for the remaining sections. Sections 4 and 5 address relevant paradigm contrasts in computational treatments of perception and action, and their implications for both biological and robotics/AI research. Sections 6 and 7 explore theoretical implications and practical applications, concluding in Section 8 with a consideration of humanoid robot consciousness (the theme of this special issue).

### 1.1 Setting Up the Framework

AIF considers a thermodynamically open, embodied, and environmentally embedded agent (see, e.g., Friston, 2009, 2010; Friston et al., 2010, 2015a,b, 2016, 2017a,b,c). In AIF, the adaptive behavior of such a “cybernetic” agent is understood to be regulated by ecologically relevant information, underpinned by a perception/action loop. Taking a broad bio-evolutionary view, AIF regards the entire embodied agent as a generative model of the organism-relevant thermodynamics of its ecological niche (see below), in that the agent is a member of a phylogenetic species that is co-stabilized with its niche. This notion encompasses the reciprocal organism/niche coevolutionary relationship (Laland et al., 2017).

During later evolutionary periods in which organisms with neural systems arise, brains come to augment the more fundamental embodied agent with a neuronal-connectivity-based extension to the generative model that handles more complex organism/niche dynamics. Thus, even when discussing PP—the human (neuronal) instantiation of active inference—the brain should be understood as “taking a back seat” to the body, serving the body by facilitating more complex coordination. Such coordination, including the dramatic niche reshaping seen in human culture, serves to co-stabilize organism and niche.

For a bacterium or a plant considered as an agent (Calvo and Friston, 2017), the embodied biological inheritance (the stable species as generative model) can be regarded as an implicit, surprise minimizing, familiarity with the niche. Many (if not all) of the earliest species inherit all the mechanisms they need for responding to and reshaping their niche, to facilitate their own survival and development. Such brainless organisms should be kept in mind whenever we “skip ahead” to the AIF description of human neural architecture—and its role in navigating the complexity of our cultural niche.^1^

### 1.2 Generative Model Basics

We next introduce the core notion of a neuronally implemented generative model. Consider, for example, a first-time visit to a university campus. Since a university is a contingent cultural entity, no part of our biological inheritance should be expected to provide us with any campus familiarity. However, if we have any earlier exposure to other universities, from visiting, reading, or hearing about them, this experience may contribute to our expectations of familiar features: we could speculatively populate any given campus with some lecture halls, administrative buildings, cafes, and so on. This mental act of populating, in other words generating, amounts to using a *generative* model of a campus (i.e., generating consequences from causes). On a first-time campus visit, such a generative model allows us to “predict” (extrapolate from the model) that there is a cafe, or, more precisely, that there is a high probability of there being a cafe, even if in actuality, there is not one there.

If we are visiting a specific campus for the first time, our generative model will be rather vague, but as we gain familiarity, we fill in more details. This process of gaining familiarity is a form of exploration, which may entail wandering, reading signs, and talking to passers-by. The exploratory process amounts to updating or nuancing our generative model for this particular campus, including specific buildings and their layout. The exploration fills in the blanks, so to speak, such that we can then exploit the model for explicit or implicit purposes, whether finding the shortest path to the cafe or aimlessly meandering on a leisurely stroll. If, when exploring the campus, every sensory impression evinces the right sort of predictions, you have effectively *inverted* your generative model. In other words, to update your model of *this* campus, it has to predict the right things in the right place at the right time. This process amounts to learning to recognize the causes “out there” in relation to their context-dependent sensory consequences, or more simply, getting a grip on how sensations are caused by attempting to predict them—and then learning how to predict in *this* context.

Thus, the explore/exploit dynamic in relation to a generative model of a niche (including any subset thereof) can be understood as a process of gaining familiarity and “leveraging” that familiarity to achieve any preferred outcome (Schwartenbeck et al., 2013). The generative model itself is augmented and developed through a broadly construed learning process that transforms neuronal networks. This developmental learning process throughout the lifespan is facilitated by, and supplements, the preceding evolutionary development of the embodied apparatus. Crucially, this learning entails something that gets quite close to conscious processing, namely a form of abductive inference that differs from standard accounts of perceptual inference, as we will see in later sections.

Significantly, in AIF, the gaining and leveraging of familiarity with respect to the generative model is not limited to agent-external (distal) phenomena. While seeing an apple in a tree is ordinarily thought of as perception (i.e., perceiving the apple or its qualities), AIF radically expands the notion of perception. In AIF, vision and the remaining four classical senses are part of exteroceptive perception, or exteroception. Beyond exteroception, however, motor-system-governed biomechanical actions, such as plucking an apple from a tree, can be perceived not only by exteroception (by sight and touch), but also by what is referred to as proprioception. Even in seemingly isolated vision, there is continuous interaction between extero- and proprioception, as visual sensing interacts with eyeball, head, and even whole-body movement. This is a fundamental move beyond PP *per se*; it acknowledges that simply making sense of sensory data is only half the problem. You also have to actively coordinate your sensory surfaces and, essentially, become the author of your own sensations. We will see later that the imperatives for the active sampling of the environment, subsequent inference, and consequent learning, all comply with the same imperative, namely to enhance familiarity or resolve uncertainty and surprise.

A further perceptual modality accounts for the sensing of hunger and related internal sensations that are not necessarily discernible through extero- or proprioception. These internal sensations are grouped together as interoception. Here, too, we must recognize the continuous interactions between interoception and the other modalities, whether in bacteria or humans. For bacteria, the generative model embodies continuous relationships between extero-, proprio-, and interoception in the form of chemotaxis and flagellar movements. For humans, when we feel an afternoon lull as a need for a snack, extero-, proprio-, and interoception interact, guiding us to the cafe to satisfy our hunger. In this light, the expanded notion of perception in AIF stretches well beyond the traditional sense of seeing the apple, in that it brings all perception and action under the same umbrella of ecologically embedded adaptive behavior.

### 1.3 Further Preliminaries

The full scope of the embodied (and optionally neuronally augmented) generative model in AIF includes the building and leveraging of familiarity with the array of interactions between extero-, proprio-, and interoception. This familiarity may be gained during the lifespan, as in human development, or it may be predominantly biologically inherited, as with bacteria. Across all cases, however, the agent seeks to bring about its preferred and familiar future (e.g., satisfying hunger) by advancing the state under its generative model, through a sequence that begins with its present state, and follows a pathway guided by (inherited or learned) familiarity. Given the exteroceptive dimension, the agent’s state can always be more comprehensively understood as the joint state of the agent/environment system.

Despite the relative simplicity of the basis of AIF—an embodied generative model with interactive modalities that facilitate agent/environment state transitions—the framework elegantly scales up from bacteria and plants to humans, even in atypical cases: a caring individual who sacrifices their own life for a preferred or expected future in which someone they rescue survives; a psychedelic drug taker who seeks a perpetually exploratory series of wild hallucinations over a more stable experience; a prisoner on a principled hunger strike who attempts to bring about a future, not of sated hunger, but of some greater social justice. In all instances, agents are interactively reducing their uncertainty in an open-ended self/world relationship (“what will happen” or “what would happen if I did that”).

This process of bringing about a preferred future is referred to (in AIF) as *active inference*, a concept that will be further fleshed out in the remaining sections. At present, it should already be clear why active inference is not continuous with earlier notions of perceptual inference, given the role of the three modalities accommodated by the generative model—especially when we consider that proprio- and interoceptive predictions change the sensory evidence for our percepts (*via* motor and autonomic reflexes, as we will see later). Arguably, even the AIF treatment of perception itself is not continuous with earlier theoretical treatments of perception, since in AIF, perception is deeply situated in the embedded context of the active agent. Moreover (as we will also see later), AIF goes beyond established paradigms critical of traditional perceptual inference such as ecological psychology, which, despite its action-oriented perspective, still exhibits a latent exteroceptive-centrism.

A final and highly significant meta-theoretical feature set of AIF—one that should appeal to humanities scholars who are wary of naturalistic and information-theoretical accounts of humanness—is that the framework inherently enshrines the fundamental uncertainty and unknowability of the future, along with the agent’s fallibility about the present and past. In addition, in contrast to superficially similar accounts, AIF markedly opposes the mechanistic to the reductive. These features will emerge more clearly throughout the paper. The next section addresses the role of the free energy principle, “the other side of the coin” of active inference.

## 2 Demystifying FEM: From Physics to Information Theory and Back Again

In this section, we use a version of Maxwell’s “demon” thought experiment to illustrate how concepts such as entropy and equilibrium link thermodynamics and information/control theory in cybernetics (e.g., Ashby), especially regarding how this link pertains to self-organization and the regulation of coupled systems. Readers already familiar with these concepts may wish to skip this section. In Section 2.1, we provide an introductory account of statistical thermodynamics and associated concepts, such as FEM, entropy, and uncertainty. We then connect these concepts to information theory and cybernetic control theory in Section 2.2. Finally, in Section 2.3, we return to thermodynamics, with an emphasis on substrate limitations for physically realized computational process models.

### 2.1 Thermodynamic FEM, Entropy, and Uncertainty

It might seem far-fetched to think that the entire universe has a direct relationship with a personal computing device. And yet, from the standpoint of thermodynamics, your laptop heats up because of the work it is doing shunting around subatomic particles, which in turn directly increases the total entropy of the universe. Of course, cosmologists have little interest in the vanishingly insignificant impact of a laptop on the universe. Scale matters a great deal in thermodynamics, because any thermodynamic system is an artificially bounded subsystem of the universe, which by stipulation, resides at the largest end of the scale. In this sense, the timescale of the universe offers the longest possible temporal trajectory, into which all other system trajectories eventually collapse.

It is a theorem in physics that the total entropy of the universe continuously increases (a corollary of the second law of thermodynamics). Thus, for any subsystem, whether a galaxy, organism, or even a laptop, if it can in any way reduce entropy within its system boundaries, this will only be for a *relatively* short time^2^ until it must yield to the entropy-increasing pressure of the universe. This relationship can be viewed as a process of maintaining a local state equilibrium at the temporary expense of a global state disequilibrium; the global state will eventually reclaim its equilibrium in the long run by overwhelming the local state.

Thermodynamic entropy can be understood as a measure of our ability to predict the position of particles within a system over a duration. This is why entropy typically increases with heat,^3^ since generally speaking, faster particle movement gives off more heat than slower movement, and faster movement leads to more-difficult-to-predict positions. Conversely, cooling slows down particles, making their positions more predictable, thereby decreasing entropy. Another way to describe the predictability of particle positions is in terms of our relative certainty about their predicted positions (in relation to the limited set of all possible positions). In this sense, higher thermodynamic entropy, greater unpredictability, and greater uncertainty are all linked to the same underlying quantity.

To bring together the notions of equilibrium states and entropy, consider a modern refrigeration unit. Its interior is kept cool by the operation of an electrical motor that gives off heat outside the unit. The entropy of the room (and indeed the universe) that houses the unit, i.e., the global equilibrium state, increases by the operation of the motor, while the cool interior, i.e., the local equilibrium state, momentarily maintains a lower entropy than the exterior. Eventually, of course, over the long run, the motor will stop, finally rewarming the unit. For keeping our drinks cool, however, it suffices to focus on the local subsystem and its corresponding timescale.

Finally, we reach the notion of FEM. In thermodynamics, particle movements count as work, and work has two main energetic effects: it uses some energy to do the work, and it releases some energy as light and/or heat. The energy available or “free” for the work is, thus, un-mysteriously referred to as free energy, in contrast to the available energy already (lawfully) dedicated to being released during the work. Returning to the above example, in a room with a refrigerator, when the fan has warmed the room air, the warm air particles have sufficient free energy to expand across the entire room. As long as the refrigerator door is closed, those particles cannot penetrate the fridge, so they only expand to occupy the room minus the fridge (a disequilibrium between the global/room and local/fridge states). However, when the fridge door is opened, the warm air particles expend their free energy by expanding into the open fridge. In this sense, they (lawfully) minimize free energy, i.e., they use the available free energy to expand across the full space, including the fridge interior. That is, through thermodynamic FEM, the global equilibrium/high entropy state of the warm room overwhelms the local equilibrium/low entropy state of the cool fridge interior.

### 2.2 FEM, Entropy, and Uncertainty in Information Theory and Cybernetics

Imagine that when we open our fridge door, a tiny demon^4^ appears, to swat away the incoming warm air particles. If it swats away a few particles at a time, it can delay the inevitable process of the fridge warming up. The more particles it can swat away, the more prolonged the delay. Better still, what if it could swat away *all* incoming particles? This would be as good as leaving the fridge door closed, as the local equilibrium of the cool interior would be maintained (at least over the short run); anything less, and the global equilibrium state (the warm room) would overwhelm the cool fridge and spoil the milk.

This demon scenario illustrates what cybernetics pioneer W. Ross Ashby (1958) termed “the law of requisite variety.” Requisite variety refers to the sufficient available responses by the local subsystem to resist the global system, such as the demon’s sufficient responses to all incoming warm air particles to maintain the cool fridge. Without requisite variety, the global equilibrium is permitted to prevail in the short run.

Now imagine the demon is working as a remote operator, controlling the positions of the cool air particles in the fridge, and maneuvering them along the plane of the door-opening to block any incoming warm air particles. This leads the particles to bounce off each other while remaining on their respective original sides of the opening, in which case the local subsystem remains thermodynamically identical before and after the onslaught of repelled particles. Significantly, the *average* thermodynamic state of the entire local subsystem is not concerned with a subset of specific particle positions. And yet, in our example, it is precisely this subset of particle positions that serve to maintain the local equilibrium. In this respect, while differing particle positions can result in thermodynamically equivalent systems, the systems would be informationally distinct, in that they reflect different organizations of the same set of particles. This brings us to Shannon (1948) information theory.

For Shannon, the distinct informational notion of entropy is borrowed from thermodynamics, as suggested by John von Neumann, who noticed the affinity between the concepts (Levine and Tribus, 1978). Shannon recognized that a set of binary switches has many possible on/off positions that can, by stipulation, be assigned any meaning. When transmitting a set of positions as a signal over a channel, noise made up of the same elements of the signal increases along the length of the channel. As this noise increases, it clouds the source signal, which in turn must be distinguished from an increasingly greater set of possible on/off switch configurations. In this sense, the location of the signal in the noise becomes increasingly uncertain.

As with particle positions in thermodynamics, the greater the ability to “predict” where the signal is within the noise, the greater the certainty. Thus, informational FEM is a reduction of uncertainty, i.e., an increased probability of picking out the relevant signal from the noise. By analogy to physics, this quantified uncertainty is termed Shannon entropy. Higher Shannon entropy reflects a greater uncertainty in picking out the relevant information, so informational FEM amounts to improving the identification of the relevant information. Technically, Shannon entropy is the expected self-information (a.k.a. *surprisal*) that (variational^5^) free energy aspires to approximate. This means that if one minimizes variational free energy at every point in time, the time average or expected surprisal is likewise minimized, thereby minimizing Shannon entropy *via* FEM.

Since the signal for Shannon is merely a particular organization of a subset of the same elements comprising the noise, the organization itself constitutes the relevant information. Of course, different organizations of the same source may be relevant under different circumstances. In Section 6.3, we will consider this sense of variable relevance in relation to the frame problem. Here, we focus on a narrow sense of relevance that builds on Ashby’s law of requisite variety.

Conant and Ashby (1970) introduced the Good Regulator Theorem. This holds that, when two systems are coupled, given requisite variety (as with our demon controller), one system can remain in its local equilibrium state (cool fridge interior), despite the pressure of the system in a global equilibrium state (warm room). Without requisite variety, the system with greater variety will overwhelm the other, subsuming it into the global equilibrium. Requisite variety can be thought of a system having sufficient control information—and response parameters—to maintain its local equilibrium (the demon re-organizing the particles). In this sense, the system is a “good regulator” of the global system and on this basis, behaves as a model of the global system. We will see later that this translates into an agent with the right sort of generative model that can generate the consequences of a variety of actions.

Crucially, using this theorem, Shannon entropy can be transformed into a sender-free construct. Specifically, for the model in local equilibrium resisting the global state, it must not only have sufficient parameters, but it must pick out the “correct” organization of elements from the global system (such that “correct” refers to the information that allows the local system to resist being overwhelmed). To illustrate the sender-free notion of Shannon entropy with the fridge example, note that there is high uncertainty concerning which subset of warm air particles and their positions will threaten the open fridge door boundary. If the demon does not continuously select and re-organize the interior particles into the “correct” (blocking) positions, the milk spoils. Informational FEM amounts to the reduction of uncertainty (sender-free Shannon entropy) concerning the warm air particles, without there being a sender transmission *per se*. This will be important later (to Gibsonians, among others) for understanding that, on the AIF conception, the environment does not *transmit* information to the ostensible sensory-receiver.

### 2.3 Design Requirements for a Brain

Finally, we return to thermodynamics, in a slightly different role. Imagine replacing our demon with an ordinary laptop running special software to perform the same role described above (identifying and blocking incoming warm air particles), with one additional constraint: the laptop must be placed inside the fridge. Lacking the demon’s thermodynamic law-defying properties, the laptop emits heat whenever it computes and controls the particle organizations. Thus, it is potentially self-defeating, since it threatens to raise the interior temperature despite keeping the outside forces at bay. Engineers could in principle redesign and reprogram the laptop to achieve efficient blocking by performing relatively few computations. A poor design might run too hot or too unreliable to be useful, while an ideal design would not overheat and block just enough particles to keep the milk cool.

This is why it is not enough to say that a thermodynamic system at local equilibrium can be a good regulator of a greater system by informational FEM alone. The local system must do thermodynamic work to be a good regulator of the greater system.^6^ Thus, the local system architecture must accomplish this work without a self-defeating heat increase (which would also increase thermodynamic entropy). This points to the fact that the means by which informational free energy is minimized must simultaneously serve to minimize thermodynamic free energy in order for the local system to maintain its equilibrium. We will see later that this theme is central to notions of efficiency, simplicity, and the elimination of redundancy that is inherent in FEM.

## 3 Evolution Through a Cybernetic Lens: Self-Organizing Systems, Embodiment, and Ecological Adaptation

Building on the previous section, we show how FEM can be used to make sense of self-organization and embodiment. We first show how physical chemistry models build on statistical thermodynamics, and how biological models build on a chemical conception of metabolic processes. We then show why physical and informational requirements are relevant to understanding embodied biological agents in relation to the coevolutionary development of species and their ecological niches.

### 3.1 Self-Organization and System Boundaries

The multiscale self-similarity of thermodynamic FEM comes into clear focus in physical chemistry. In a chemical system, predicting the behavior of individual particles can be intractable, but we can use the same mathematical models for particle aggregations as for individual particles. A transparent example of this is the process of crystal formation, called nucleation (Auer and Frenkel, 2001). In a pool of solute, many particles are distributed throughout. Typically, the behavior of the liquid is such that, for the particles to minimize (thermodynamic) free energy, they simply follow the liquid flow patterns (i.e., the paths of least resistance, in other words, the least surprising trajectories). However, if the right subset of particles comes into proximity, their thermodynamic FEM will in fact lead them to aggregate together. This particle aggregation will continue to swirl around in the pool and, at various points, more particles will begin to follow a pathway that affords greater FEM by joining the aggregation than by swirling around apart from it. The aggregation becomes the nucleus of an emergent crystal formation, which reaches a critical tipping point that leads an increasing number of particles to join up with it in a crystalline structural arrangement—all this mandated by simply following the path of least resistance at each point in time.

In virtue of this pattern, the crystal is distinct from the pool: it is an emergent self-organizing system with sharp boundaries. Specifically, the crystal is a free-energy-minimized molecular arrangement which has a lower-entropy local equilibrium than the contrasting higher-entropy global equilibrium of the pool. Of course, the crystal is merely an inanimate rock. Consider, however, another equivalent self-organizing criticality system, a forest fire (Drossel and Schwabl, 1992; Malamud et al., 1998). There is a critical tipping point at which the chemical process of the fire gains the capacity to spread according to a pattern of available fuel, to continue the chemical catalytic process. The forest fire, like the crystal, has clear system boundaries that emerge. Unlike the crystal, however, the nature of the fire’s metabolic process means its system boundaries will not be maintained without additional fuel, in which case the fire will “die out.”

This metaphor of fire “dying” aptly reflects the fact that biological systems also exhibit self-organized criticality, with a parallel metabolism that demands fuel to maintain system boundaries. A bacterium must obtain fuel from beyond its system boundaries to burn within those boundaries, in order to maintain them. Hence, there is a direct continuity and self-similarity across self-organizing aggregations-as-embodied systems from physics to chemistry to biology (Sengupta et al., 2013; Friston et al., 2015a,b; cf. Chemero, 2008; Bruineberg and Rietveld, 2014).

### 3.2 Ecological Context

At the biological level of description, the theoretical vantage point of ecology becomes relevant to understanding how organisms keep a positive balance in their metabolic bank account, so to speak. The cybernetic evolutionary lens described above reveals the connection of the embodied organism to the AIF notion of a generative model. Specifically, the embodied agent has a “do or die” to-do list to maintain its system boundaries, or more comprehensively, to survive and thrive. This list includes the agent obtaining fuel from its niche (to sustain its metabolism), avoiding active existential threats (e.g., predators), and also remaining within its embodied-apparatus-relative niche boundaries by not being a fish out of water, a land mammal falling down a ravine, or indeed any organism exceeding atmospheric thresholds of high and low temperatures and surface pressures.

Broadly, this set of agentive processes can be understood as an active engagement in a homeostasis/allostasis dynamic (Pezzulo et al., 2015), which more broadly still, can be regarded as adaptive behavior. For adaptive behavior to succeed, that is, for the organism to survive and thrive, it must have inborn and/or acquired familiarity with itself and its niche. In other words, the agent must be able to act on control information concerning its self/niche relationship (Friston, 2014). This control information can be understood as embodied system-boundary-internal adaptive behavioral guidance information, with the sole requirement that it is good enough for facilitating the agent’s ability to survive and thrive, akin to satisficing (Simon, 1957).

Notice, however, that despite foregrounding the importance of boundaries, the picture is one in which living organizations are themselves changeable in ways that minimize the free energy of an evolving process (see, e.g., Clark, 2017). Notice also that, despite the sometimes-grim connotations of cybernetics and control theory, the notion of “control” is here synonymous with regulation, in the sense that you control, i.e., regulate, your own appetite simply by eating. In this sense, for the organism to be a good regulator, it must have a satisficing degree of certainty about itself and its niche to pick out what is relevant to its “to-do” list, such as responding to perceived hunger or danger, e.g., by seeking food or shelter. In logically equivalent terms, the agent must reduce its uncertainty, i.e., minimize (variational) free energy for a thermodynamic payoff.

To achieve this FEM, on an evolutionary timescale, organisms may mutate and potentially become an embodied generative model of a new niche. On a lifespan timescale, they may explore their niche to learn its contours, find new sources of sustenance and shelter, and new threats to avoid, i.e., augment their inborn generative model. In the interplay of evolutionary and lifespan trajectories, organisms transform their niches, bringing about higher-certainty correspondences to some aspects of their embodied generative model (e.g., tunneling underground to cushion light sensitivity). Indeed, some perspectives in theoretical biology speak to evolution itself as a FEM process, for instance, generalizing Darwinian processes as physical implementations of Bayesian inference (Frank, 2012; Lammert et al., 2012; Campbell, 2016).

Early lineages of organisms including bacteria and plants respond to self and environmental regularities even without a neural system, whereas later lineages including humans have the further support of a neural system to respond to more statistically complex regularities. Such complexity is reflected by increasing neuronal connectivity throughout the evolution of stable species. The ability to identify regularities in control information that reflect (self and niche) thermodynamic regularities can thus be viewed as an ecological adaptation requirement. By attaining effectively low uncertainty concerning adaptively relevant niche information—that is, by continuously minimizing (variational) free energy—the embodied agent is able to maintain a stable local (thermodynamic) equilibrium. The agent thereby resists the potentially overwhelming pressures of the environmental global equilibrium (the second law of thermodynamics) for the limited duration of its lifespan.

### 3.3 Complexity and Spatiotemporal Integration

Given our account thus far, it should be clear why, from a “good regulator” perspective, the more informationally complex the niche, the more complex the embodied (and eventually brain-augmented) generative model must be to facilitate effective adaptive behavior. The basic reflexive behavior, from bacterial chemotaxis to some plant and even insect behaviors, indicates that the preponderance of adaptive “work” can be done at a deeply embodied level, with low-level connectivity requirements (see, e.g., Mann et al., 2017). This is why for Gibsonian ecological psychology and Brooksian robotics, the bulk of relevant regularities are regarded as being wholly external to the embodied (natural or artificial) agent.

However, the theoretical framing device positing that “the world is its own best model” (Brooks, 1999) ultimately does not scale up to account for more complex agent/niche interaction dynamics. From the AIF perspective, it might be said simply that the world is its own best *world*, while the embodied agent itself is the best model of those aspects of the world relevant to its surviving and thriving—a familiar econiche that it has largely constructed for itself (Laland et al., 2017). Arguably, in relation to evolutionary natural selection pressure arising from niche saturation, mutants will only survive to stabilize as a new species under one of two conditions: expanding into a new niche that is spatially beyond the saturated niche, or expanding into one that is spatially coextensive with it, but presents a different set of relevant regularities (see Ito and Ikegami, 2006). In the latter case, the corresponding increasing informational complexity of the niche plausibly relates to increasing organismic complexity (coevolution).^7^ Once neural systems emerge, this coevolutionary pattern continues with increasing neuronal connectivity (Yaeger, 2009; see also Seth and Edelman, 2004; Yaeger and Sporns, 2006; Yaeger, 2013).

Continuing with this account, a significant meta-theoretical feature of AIF can be noted, namely, that the human individual is re-contextualized as emerging naturally from the social group. There has been increasing interest in socially grounded neuroscience (e.g., Dumas et al., 2010; Dumas, 2011) and social robotics (Leite et al., 2013). Yet, some accounts largely consistent with AIF (e.g., Butz, 2016) only consider the social as an afterthought to the individual. Under the above considerations, however, given the upper bound on individual brain capabilities from a thermodynamic perspective, for humans to stabilize as a species, social cooperation offers the greatest advantage for establishing an adequate niche to sustain a stable population (see Yoshida et al., 2008). Indeed, identifying evolutionary stable strategies in multi-agent games, within AIF, can lead to some counterintuitive yet compelling conclusions, particularly in terms of the degree of sophistication agents require in relation to others (see Devaine et al., 2014).

At the same time, as human culture emerges, introducing even greater niche complexity, the very same cooperative distributed information dynamics can lead to inherent difficulties. It is intrinsic to the underlying mathematical model of AIF that an apparatus which evolved for reducing uncertainty is equally sufficient for *increasing* uncertainty under particular circumstances. This is evident in social misunderstandings, such as mistaking the attributed motivation of a facial expression (Clark, 2015b, Section 2.9). The potential for the system to backfire, so to speak, is a consequence of the fact that human niche complexity includes social and cultural relationships, artifacts, language, and so on, which corresponds to substantially more complex neuronal connectivity in humans as compared to our evolutionary predecessors (Street et al., 2017). Even within human groups, a narrower, more predominantly physical, interpersonal local niche engagement (e.g., a stag hunt) requires considerably less informational complexity than the vast distributed neural/environmental information dynamics across a broad integrated physical and sociocultural niche. In the latter, agents face a greater challenge in leveraging more radically limited partial information (Ramamoorthy et al., 2012).

As neural complexity increases on an evolutionary timescale, the AIF model of the neural architecture is described in terms of an increasing number of interconnected hierarchical layers. These layers facilitate more extended spatiotemporal integration, with a growing set of nested local scales of time and space, ranging from the immediacy of the reflex arc, to ecologically situated behavior, to the lifespan. For instance, a beaver building a dam must be able to handle more extended time and space than a bacterium. Primates (including humans) exhibit nested spatiotemporal integration when interactively engaged in a dynamic situation or observing a visual sequence, as do humans when following along with speech or writing by integrating syllables into words, words into sentences, and sentences into a narrative (Hasson et al., 2008; Kiebel et al., 2008; Chen et al., 2015; Friston et al., 2017c; Yeshurun et al., 2017). This complex nesting, which has been implemented in robotics (Modayil et al., 2014), corresponds to a neural architecture that instantiates active inference in humans as PP, with growing empirical evidence of neurobiological substrate correspondences (Friston and Buzsáki, 2016; see also Clark, 2013, 2015b).

## 4 Unveiling the World, Upending the Input/Output Model of Perception (and Action)

With a focus on brains, this section shows how AIF upends the input/output model of perception (and action) still prevalent in embodied cognition and ecological psychology research, and perhaps even more prominently so in robotics/AI. As the full implications of this upending unfold, two major theoretical problems—the inverse problem and the frame problem—are revealed to be artifacts of the input/output model, such that AIF does not merely solve, but in fact dissolves these problems. Moreover, the philosophical concern raised against PP (and by extension, AIF), namely, that it entails or implies a solipsistic agent, hermetically sealed off from the world by an evidentiary boundary (or “veil”), is shown to be unfounded.

### 4.1 The Poverty of Indirect and Direct Perception

Is the embodied generative model stuck behind an “evidentiary boundary” (or “veil”), with no direct access to an outer world that is merely inferred? This is the notion of indirect perception that Hohwy (2013, 2016) advocates (cf. Clark, 2016). What Hohwy misses is a relevant distinction between phenomenal sensation and control information (elaborated in this section). Following the AIF account outlined above, control information provides the possibility for the agent being a good regulator. However, this remains distinct from phenomenal sensation of the world. At the same time, phenomenal sensation can itself be harvested for control information, in addition to information beneath the awareness threshold (Kang et al., 2017).^8^

Consider, for example, a video conference call apparatus. In an efficient design, the data flowing from one call participant to another will serve two simultaneous roles: a qualitative (content-relevant) role, in that the data underpin the audiovisual streams by which the parties can converse; and, at the same time, the data will serve a quantitative (content-irrelevant) role as control information, in that the data transfer rate will modulate the audiovisual resolution to compensate for bandwidth variation. In a parallel sense, in AIF, there is direct thermodynamic engagement between the agent’s sensory surfaces and the world. This is precisely why we wear special glasses to view an eclipse, or earplugs at a loud concert: the direct engagement can be so powerful as to be biologically destructive. At lower intensities, light and sound contribute to a variety of enjoyable phenomenal sensations, and yet, they serve a dual role as control information. Under situations of acute existential threat, the control information may be the only relevant signal, whereas under presumed existential comfort (e.g., at the cinema), the control information may be largely dampened while (by cultural convention) phenomenal sensations are experienced for their own sake. Most quotidian cases lie somewhere in between these two extremes, such as eating to satisfy hunger while simultaneously savoring the sensory delights.

Given the broadly Helmholtzian inference tradition that Hohwy draws on, it is notable that this is precisely the kind of inference that Gibson (1979/1986) criticizes in his elaboration of ecological psychology, finding fault in theories in which “the outer world is deduced”:

The traditional theories of perception take it for granted that what we see now, present experience, is the sensory basis of our perception of the environment and that what we have seen up to now, past experience, is added to it (pp. 251ff.).

This critique motivates Gibson’s positive account of “direct perception,” also referred to as “information pickup” (Gibson, 1979/1986, pp. 147ff.). And yet, upon closer analysis, his positive account results in many of the same theoretical shortcomings as the inferential model he criticizes, as we will see below (cf. Fodor and Pylyshyn, 2002).

Both Helmholtz and Gibson ultimately inherit the same problems from the classical input/output model of perception. What Gibson criticizes in traditional inferential theories is the notion of passive input, which he replaces with active input—but it is still input! The active component in Gibson hints at the significance of proprioception, but ultimately, he assigns it an exteroceptive-centric role (Gibson, 1979/1986, p. 141). To make this argument, we first present the classical input/output model shared by computational perceptual theory (conventional in biology and robotics/AI) and contrast it with AIF.

### 4.2 Classical Computation vs. Active Inference

The classical input/output model of perception (and action) is the predominant model used in psychological, neuroscientific, and robotic explanations; this model also typically underlies the notion of neural computation and information processing, and it is ripe for retirement (Clark, 2014). AIF implies a vastly different conception of the relationship between perception, action, and the world, that also points to a different sense of computation and indeed perception itself. To understand AIF’s ontological commitments and implications for perceptual theory generally, and for robotics/AI, we must examine the assumptions and implications of the predominant model.

The basic elements and processes of the classical/computational model can be generalized as follows: un-encoded (“raw”) data from the environment (“world”) is selectively sampled by the agent and encoded as input (“reading” the raw data). This raw data input, once encoded into the system, is then processed (beginning with “early perception”). This processing chain produces a decoded output, terminating as a percept (and potentially entering into a secondary stage related to concepts). After this discrete stage, as this story goes, an executive controller may then retrieve the percept (or concept) from storage and engage it in further action-relevant computations or reflexively issue a reactive action command.

Significantly, two major problems arise as mere artifacts of this model—the inverse problem and the frame problem. Both have given rise to countless accounts of how to bypass or solve them. Most famously, Marr (1982) produces a highly influential and elaborate account of how to solve the inverse problem, to get from the input stage to meaningful experience of the world. His solution comprises an elaborate series of “early” perceptual processing stages for disambiguating apparent equivalencies, implemented in subsequent decades of computer vision research. Marr was in part responding critically to Gibson’s account, although some readings offer a middle ground between the two theories (Ullman, 1980; see also Shagrir, 2010). Gibson (1979/1986) and later analysts of ecological psychology argue that the inverse problem is bypassed without appealing to the kinds of processes Marr introduces (e.g., Hatfield, 2003; Chemero, 2009; Orlandi, 2017), for instance, by bodily movements (exploring or swaying) that reveal constant proportions in three-dimensional situatedness, in contrast to two-dimensional sources of optical projections. Like Marr, however, these ecological accounts still treat (what is regarded as) exteroceptive input as primary, even when the necessity of proprioceptive coupling is acknowledged.

Those who accept the classical/computational input/output model of perception must also face the frame problem (McCarthy and Hayes, 1969; Minsky, 1974), which can be generalized as a problem of knowing when and what raw sampling is needed for updating beliefs about the world (e.g., in relation to an isolated local action that only modifies a small subset of the environment^9^). It also concerns how to handle an input encoding from one context following a change of context. Thus, the frame problem is also known as the “relevance” (or “significance”) problem, based on the premise that there is no obvious means of ascertaining what is cognitively relevant or significant under changing circumstances. The frame problem has led to elaborate logic-based solutions (Shanahan, 1997) and critical accounts of robotic AI based on embodied phenomenological philosophy (Dreyfus, 1992, 2007; cf. Wheeler, 2008).

### 4.3 Upending the Input/Output Model of Perception (and Action)

Building on the previous sections, we briefly show how AIF re-arranges the picture to dispense with the classical/computational model of input and output. Recall that above, we noted that there is direct thermodynamic engagement between the agent’s sensory surfaces and the world, which requires protection from high intensities (e.g., earplugs at a loud concert). For an intuitive example of lower intensity engagement, consider a game of tennis. It would take some mental gymnastics to make sense of the idea that an arm is input to a racket, and a racket input to a ball—on this view, what would count as output? Instead, using basic physics, we regard the action of hitting the ball as a transfer of energy, from the arm to the racket to the ball. This same sense of thermodynamic energy transfer occurs between an organism’s environmental niche and its sensory surfaces.

In AIF, the embodied agent learns the regularities of the sensory surface perturbations, much like what Gibson (1979/1986) refers to as invariants. Moving beyond Gibson, in AIF, the invariants extend across interactive regularities in extero-, proprio-, and interoception, in the form of the generative hierarchical model. The more regular covariance that is learned, such as how invariant proprioceptive hand-grasping patterns covary with invariant racket-swinging, ball-hitting patterns, the more reliable the generative model is as control information across a variety of conditions to which the model is adapted (see Kruschke, 2008). In PP, this adaptive process proceeds by a feedback loop with prediction error, i.e., minimizing prediction error amounts to adapting the generative model to the present conditions (Clark, 2013, 2015a,b).

The continuous embedding in the niche, which the agent explores to learn the covariance regularities, allows the agent to develop and update the generative model (akin to Gibson’s notions of “tuning” and “resonance”). This goes beyond the exteroceptive-centric notion that minor proprioceptive alterations bypass the inverse problem. In AIF, the generative model links all reliably invariant information in a deeply situated way, such that perception and action enable the embodied agent to propel itself through a temporal succession of generative model modulations, for instance, approaching a distal food source to eventually alleviate hunger.

Under such situated embedding, the frame problem never presents itself, because the relevant aspects of the niche are thermodynamic perturbations, while engagement with the niche is facilitated by continuous control information. In the preponderance of ecologically valid conditions, there is never a temporally suspended slice of un-embedded input to be processed, nor is there an isolated (i.e., non-deeply situated) encounter with an exteroceptive input stimulus that is lightly probed through proprioception. That is, in real-world embodied and embedded cognition, there are no disconnected moments of perception of the world, since the world wholly envelops the agent throughout its lifespan. (We return to the frame problem in Section 6.3.)

Ambiguities arising from thermodynamically relevant niche details can indeed fail to be disambiguated, as they do during contrived experiments and illusions. However, in AIF, ambiguity is not an “early perception” input processing challenge, but rather a matter of the precision-weighting of layers of the hierarchical architecture (Friston, 2008). Many situated perceptual ambiguities can be accommodated by the precision-weighting of higher or lower layers: higher layers provide broad continuities to previous situations, such that ambiguities closer to the sensory surface can be ignored or recognized as illusory (as when the magician’s assistant seems to disappear into thin air), while ambiguities at higher levels can be suspended pending further lower-level evidence (as when it is unclear if a friend entered the theater or joined the crowd outside). In addition, perceptual disambiguation is facilitated by the nested multiscale dynamics described above (Brascamp et al., 2008).

## 5 Gibson Reconfigured: Beyond Re-Description

Notably, AIF carries forward Gibson’s core critique of his behaviorist and cognitivist predecessors; however, AIF also addresses the fundamental inadequacies of his positive account, as we illustrate in this section. We begin with an initial re-description or translation of some Gibsonian concepts into AIF. At relevant points throughout, we also highlight connections to robotics.

### 5.1 Initial Mappings

Recall from above Gibson’s objection to theories (e.g., Helmholtz’s) in which the present perception of the world is inferred by an additive process that uses the past (memory) to supplement missing details. Here, a technical clarification will be useful to distinguish traditional perceptual inference from AIF/PP. Shortly, we will flesh out what the actual process of “active inference” entails, but for now, it can be stated that in PP, the prediction of the present is fundamentally non-inferential in the traditional sense (see below for the specialized sense of surprisal-reducing model inference). Instead, perceiving the present is facilitated by an extrapolation from the environmentally embedded generative model. The model develops through biological inheritance and lifespan experience, based entirely on invariant covariance of modalities from past interactions.

Perception in AIF is thus not an additive process, but a generative one, which matters here for an important class of cases, namely, those in the cultural (as opposed to natural) domain. The cultural domain has physically bound cases with no natural equivalent, such as the operation of a door with a doorknob. We see many naturalistic examples in Gibson’s writings, concerning, e.g., tunnels (which may occur in nature), but he also wishes to extend his theory to the human cultural environment (Gibson, 1966). Moreover, he wants to allow for a concept of learning (at best, coarsely defined), while simultaneously objecting to a model of mental storage and retrieval (Gibson, 1979/1986). How then, should it be possible to learn how a doorknob works such that “direct perception” of one (*via* ambient optical arrays) is at once the perception of a means for opening the door, without any specified mechanism for establishing this correspondence? If the correspondence is merely a conditioned association, then how can he avoid the claim (as he intends) that past experience is added to the present?

Despite Gibson’s professed aversion to computation and traditional perceptual inference, the deeper problem here is that his theory recapitulates and is thus still bound by the classical/computational input/output model (cf. Bickhard and Richie, 1983). To better understand this issue, we must turn to his concept of affordances. For clarity, we will first establish how AIF re-describes aspects of Gibson’s ecological framework in terms of the generative model.

In some AIF contexts (FitzGerald et al., 2014), it is more useful to treat the generative model as a model *space* populated with an ensemble of plausible generative models. For instance, consider a proprioceptive model of hand configurations: grasping, wrist rotation, peripersonal reach, and so on. To be clear, this sense of generative model is not an imagistic mental representation, but rather, a mathematical model of a set of invariant synaptic firing patterns that reliably correspond to bodily movements. These proprioceptive models (subsets of the complete generative model) are equivalent to Gibson’s notion of organismic capacities. Within the model space, there are also exteroceptive models that reliably correspond to sensory perturbations caused by, e.g., trees and branches, doors and doorknobs, and so on, which relative to proprioception, re-describe Gibson’s notion of environmental action opportunities (a branch affords climbing a tree, relative to the bodies of certain organisms). In his theory of affordances, Gibson also notes the relevance of the organism’s wants and needs. These are incorporated into AIF as prior beliefs or preferences constituted by the generative model. Key among these are the priors over interoceptive predictions, by which we reliably come to recognize internal sensations such as hunger, fatigue, lack of fresh air, and so on (Seth et al., 2012).

Each of these models interact within a hierarchical model space, such that single modality invariants intersect and interact with each other, resulting in invariant covariance relationships: (interoceptive) hunger is reduced by eating fruit from a tree, which can be (exteroceptively) seen and (proprioceptively) reached by climbing branches. In a cultural context, the (interoceptive) need for fresh air can be met by (exteroceptively) transitioning from indoors to outdoors, as facilitated by a (proprioceptive) action sequence involving turning the doorknob and walking out of the room. The action sequence itself can be further broken down, in that even the doorknob interaction is a result of invariant covariance between exteroceptive control information and proprioceptive reaching, grasping, and turning; this principle has been successfully robotically simulated (Pio-Lopez et al., 2016). In brief, AIF offers a fundamentally embodied and embedded account of situated perception and action, rather than an exteroceptive-centric input/output model. The latter requires traditional perceptual inference based on early (perception) input processing of an impoverished stimulus; or, as Gibson has it, such inference is replaced by a woefully underspecified “direct perception” mechanism that fails to explain learned cultural affordances.

To summarize this initial re-description of Gibson’s framework in AIF, and more importantly, the underlying shift in emphasis, we have seen that Gibson’s affordances concern the perception of (a) environmentally specified information as action opportunities in relation to the organism’s (b) embodied capacities and (c) needs and wants. In AIF, all three are integrated into the embodied (and neuronally augmented) hierarchical generative model, with correspondences to Gibson in terms of (a) exteroception, (b) proprioception, and (c) interoception. This allows us to make sense of a common ecologically valid scenario, such as the interoceptive need for fresh air, and the extero- and proprioceptive interactions that lead to turning the doorknob, opening the door, and walking outside. We are now in a position to flesh out what “active inference” itself refers to, which requires the introduction of a specialized concept: policies.

### 5.2 Affordances and Policies

The notion of policies highlights how the generative model can be temporally deployed over possible future states. Once this is understood, the full implications of embedded spatiotemporal nesting and its relationship to agent/environment dynamics can be brought into view. Policies are means of transitioning between states of the generative model, which can only be in one (actualized) state at a time.^10^ The conventional sense of actions (e.g., reaching for the doorknob) “fall out” of policies, as we will see next.

A theoretician seeking to define a policy in propositional terms might define one (in the following example) as “go outside to get fresh air.” The underpinnings of the policy are in effect a possible transition between two states of the generative model: the current state (at time t_0_) and a preferred future state (at time t_1_). At t_0_, the agent is inside a room with a door to the outside. In the exteroceptive modality (in addition to phenomenal sensation), there is control information present concerning walls, doors, doorknob mechanisms, and so on. There is also proprioceptive (control) information available concerning, e.g., hand-grasping and leg-walking abilities. In the interoceptive modality, there is information concerning a sensed lack of fresh air and its presumed contribution to fatigue.

In this case, the preferred future outcome is having fatigue alleviated by getting fresh air. This would mean that if this outcome were attained, at t_1_, the generative model would be altered, such that the exteroceptive information would pertain to an outdoor rather than indoor scene, and the interoceptive information would pertain to breathing fresh rather than stale air. To realize the preferred outcome, the agent *actively infers* the (t_0_ to t_1_ state transition) policy. Working backwards in a sense, to facilitate this transition, a series of actions “fall out,” unfolding without requiring the planning of a sequence of action commands (Adams et al., 2013), in stark contrast to the robotics paradigm of sense-plan-act. Instead, the reliable covariance with proprioception and the other modalities of the generative model leads to reaching, grasping, and turning the doorknob, to open the door, to walk outside, to get fresh air, given that this set of covariances has been empirically established (i.e., learned).

The bottom line here is that if an agent entertains a generative model of the future, the agent must have beliefs (i.e., expectations) about future or counterfactual states under each allowable policy. Put simply, we have in mind here an agent whose generative model transcends the present and is continuously predicting the future (and past). Crucially, each prediction—at different times in the future—is subject to the same policy-dependent transition probabilities as apply to the here and now, thereby “connecting the dots” in a path to preferred and familiar outcomes. On this view, the present simply provides sensory evidence for one of several (counterfactual) paths into the future, where the path (or policy) with the greatest evidence gets to determine the next action. Notice again how we return to the path of least resistance or minimum (expected) free energy (i.e., maximizing model evidence over possible pathways).

Through a continuous series of perception/action loops, the embodied agent remains in open exchange with the world by actively probing its environment (Kruschke, 2008) and leveraging the control information of the generative model to alter the thermodynamic substrate (its physical position and condition). Even Gibson could not object to this sense of inference: there can be no “direct perception” of the future! Here, however, is where the uncertainty and unknowability of the future can be understood as a feature of AIF that is lacking in ecological psychology, namely, concerning *conditional* future outcomes. Even on the most charitable reading of Gibson, assuming we can explain (without magic) that one could “directly” perceive that “the doorknob affords opening the door” based on the ambient optical array, conventional affordance theory is left stranded in the face of an invisibly locked or broken doorknob. That is, when the doorknob fails to open the door, the exteroceptively ascertained ambient optical array remains identical before and after the attempt. Thus, within Gibson’s framework, the doorknob forcibly remains an apparent affordance even with prior information that it does not open the door in this case. In such ecologically valid scenarios commonly faced by human cognition, it is a severe meta-theoretical weakness if they cannot be adequately addressed.

In contrast to ecological psychology, AIF elegantly handles conditional outcomes in terms of probabilities. This is why it uses a Bayesian model of neural processing, given that empirical priors derived from experience influence the generative model computations of probability,^11^ a significantly different sense of computation than that used in input/output model descriptions (which hold that sampled input is computed/processed). Reconfigured by AIF, a typical affordance is merely a high likelihood, such that “affords” amounts to “offers a relatively sure bet.” Thus, “the doorknob affords opening the door” is more accurately rendered as “the doorknob offers a relatively sure bet for opening the door,” thereby accounting for the conditional outcomes in which the doorknob is locked or broken, unknowable by exteroception alone. In addition, when a source of information indicates a locked or broken state (such as a performed or observed attempt to open it, or by word of mouth), the doorknob ceases to be an apparent affordance, since it no longer offers the agent a relatively sure bet for opening the door, despite the fact that the ambient optical array is unaltered.

AIF is consistent with the view that “affordances are relations.” More precisely, “affordances must belong to animal/environment systems, not just the environment,” in that perceiving affordances is perceiving “the relation between the perceiver and the environment” (Chemero, 2003, pp. 185–6; see also Chemero, 2008). By adding the extended temporal dimension of AIF, the affordance relationality can be further understood as being between a presently given agent/environment relational state and probable future agent/environment relational states.

This move also allows AIF to account for conditions in a more distant future, such as dinner plans next week, which some theorists view as beyond the scope of ecological (and enactive) explanation. Here, such planning ability is seamlessly accounted for in the process of active inference. The plan sets into motion a series of intermediary interactions (actively inferred state transition policies) that propel the embodied agent toward the preferred future outcome. These interactions are based on experience and are, thus, deemed reliable (in a satisficing sense) with reasonably high probability, while (simultaneously) suggesting a low-probability capacity to fail. Put simply, all I need to do to determine my next action is to choose the most probable action under the prior belief: “I will not miss next week’s dinner party.” This prior belief generates a hierarchical cascade of empirical priors, each providing contextual guidance to accumulate the sensory evidence for the particular path I am pursuing. If everything goes well, this path would end successfully with arrival at the dinner party. Note that not only is there a deep generative model in relation to time in play here (Dehaene et al., 2015), there is also a hierarchical depth in terms of short and long-term policies, i.e., trajectories of states (see Friston et al., 2017c).

### 5.3 Free Energy, Revisited

What does all this have to do with the free energy principle? The policies the agent infers, as transitions from present to preferred future state, are those that minimize (variational) free energy expected on actualizing the preferred future state. This contextualizes the notion of reward motivations (that policies increase expected future reward) and even problem-solving itself, in that the reward or the solutions are part of the preferred future outcome as viewed from a present state (Friston et al., 2009, 2010; Friston, 2011; cf. Newell et al., 1959). Technically speaking, the expected free energy ensures that the prior probability of a policy maximizes reward (i.e., prior preferences) in the future, as in machine learning, under the constraint that it also minimizes uncertainty and ambiguity. Moreover, in the agent’s relationship to the niche, expected free energy is minimized—uncertainty or disequilibrium is reduced (see Sections 2 and 3)—as the agent strives to select the relevant control information in the face of the densely rich informational environment (high Shannon entropy). This is an important point which takes affordances into the epistemic realm.

In other words, by trying to infer the FEM path of least resistance into the future (even for a challenging task), there is a necessary component of uncertainty that combines with prior preferences to determine the best policy. This means that the most probable policies or paths are those that resolve uncertainty when navigating the lived world (Berlyne, 1950; Schmidhuber, 2006; Baranes and Oudeyer, 2009; Still and Precup, 2012; Barto et al., 2013; Moulin and Souchay, 2015). To achieve this, agents engage in some interactions that serve an epistemic rather than pragmatic purpose, i.e., epistemic actions (Kirsh and Maglio, 1994). In AIF, we can place such epistemic actions in the general context of physical or mental epistemic foraging (Pezzulo, 2017), and further specify what facilitates such epistemic actions, namely, *epistemic affordances*. The latter concept brings with it the notions of salience—epistemic affordances that will reduce uncertainty about future states of the world—and novelty—epistemic affordances that will reduce uncertainty about the contingencies or parameters of my generative model. (The next section furthers this account of affordances.)

In summary, one’s preferred future state is realized by exploiting high likelihoods in the sequence of state transitions of the generative model that underpins the agent/environment relationship (e.g., my relatively high certainty that my hand turns a doorknob, which opens a doorway, which I can walk through to get outside, to get fresh air, and to alleviate my fatigue). Exploiting high likelihoods refers to the probabilistic Bayesian decision-making computations that play out on a dynamic, neurobiological substrate (Pezzulo et al., 2015). In this context, it can be said that *local minima of uncertainty* (in the projected model state transitions) provide the critical points that can be leveraged to facilitate a preferred future (or avoid an undesired future). At the ecological “behavior” scale (policies), these local minima provide a comprehensive re-description of affordances that unites the exteroceptive with the proprio- and interoceptive dimensions (Pezzulo and Cisek, 2016). They also generalize to the sub-ecological “action” scale, as reflex arcs, grounded in the physics of nerve electricity (Friston et al., 2010; Sengupta et al., 2013), and the supra-ecological “activity” scale, as extended active and resting states, grounded in physiological homeostasis/allostasis dynamics (Ashourvan et al., 2017).

## 6 Skating Uncertainty: Generalized Affordance Theory, Skilled Expertise, and the Frame Problem

This section considers how local minima of uncertainty in the projected temporal sequence of generative model states serve to unify developmental theory and the underspecified (by Gibson) notion of learned affordances. We then show concrete applications in skilled practical and cultural activities. Finally, drawing on robotics studies, we connect spatiotemporal nesting and agent/environment dynamics to adaptive policy reuse.

### 6.1 Generalized Affordance Theory

Here, we generalize affordances to every available reliable regularity in the agent/environment relationship, including basic objects. While this level of generality may seem meta-theoretically undesirable, it is worth bearing in mind that Gibson extended affordances to this high level of generality in explaining that air affords breathing, the ground affords standing on, cliffs are negative affordances for bipedal locomotion, and so on (Gibson, 1979/1986). On our account, affordances encompass the entirety of intuitive physics (see Clark, 2016).

As Franz and Triesch (2010) argue, a number of purported Gestalt percepts have only been considered in relatively late periods of individual (lifespan) human development, as even within the first several months after birth, there is a tremendous amount of densely rich environmental information encountered. The inborn apparatus (as suggested by AIF) for discerning regular covariance and leveraging that in situated activity can be computationally simulated with only a limited construct that yields a number of Gestalt-like phenomena. The limited construct—foreground and background differentiation—is a minimal mechanism that would be plausibly selected for on an evolutionary timescale.

In addition, there appears to be another plausibly selected for (inborn) minimal mechanism for differentiating inanimate from animate entities, with the latter possibly extending to finer-grained differentiations between conspecifics and other animals. There is evidence of this mechanism in brain scans of primates (Sliwa and Freiwald, 2017) and human infants (de Haan and Nelson, 1999, Southgate et al., 2008), and from human *in utero* behavioral experiments (Reid et al., 2017). This mechanism would plausibly underpin the fundamentality of social cooperation to human cognition (Barrett et al., 2010, Cortina and Liotti, 2010); a related point has been made about language, noting the fundamentality of dialog from which monolog is derived (Pickering and Garrod, 2004).

The above suggests that early developmental learning proceeds through interactive exploration (Stahl and Feigenson, 2015), which makes possible a high-level generative model of intuitive physics that augments inborn capacities with empirical priors. This is especially evident from the gradual development of coordinated bodily movement, ranging from basic crawling, walking, and stacking blocks, also explored in robotics (Pierce and Kuipers, 1997, Modayil and Kuipers, 2008, Ugur et al., 2011, 2012), all the way up to more elaborate activities such as interpersonally coordinated dancing and playing sports (Boyer and Barrett, 2005). Based on reliable covariance from empirical priors and inborn minimal mechanisms for differentiating foreground and conspecifics, the present state and future projections of the generative model facilitate (*via* actively inferred policies) the realization of preferred outcomes through the exploitation of local minima of uncertainty, i.e., generalized affordances. It is in this context that epistemic affordances play a key role and can be associated with intrinsic motivation, exploration, “motor babbling” and artificial curiosity in developmental neurorobotics (Schmidhuber, 2006, Baranes and Oudeyer, 2009). Put simply, being compelled to pursue FEM, uncertainty-reducing epistemically enriched policies ensure that agents quickly come to discover “what would happen if I did that.”

Consider an example that works both literally and as a broad analogy to this generalized affordance process: the crossing of a roaring rapids *via* stepping stones. The rapids are in constant flux, but the fluctuations of the water also momentarily expose surface regions of the stones. In this sense, despite the high uncertainty brought about by the flux, the overlapping exposed surface regions for each stepping stone provide stable points—local minima of uncertainty. These local minima facilitate crossing the river, by which the preferred outcome of reaching the opposite bank is realized. In a literal sense, the stones are clearly conventional Gibsonian affordances, presented here as local minima of uncertainty in sequential states of the generative model. Analogically, the roaring rapids correspond to the general sensory flux of thermodynamic surface impingements, and the stepping stones correspond to any reliably invariant multimodal covariance established by empirical model updating. This sense of local minima also suggests a formal correspondence to the basins of attraction in neurodynamics (Freeman, 2012).

### 6.2 Skilled Expertise

By considering affordances in this light, we can demonstrate how affordance theory relates to arguments about skilled expertise from the perspective of phenomenological philosophy. The latter argues for the central role of embodiment as the basis of skilled expertise, in contrast to some conventional theories that view expertise in terms of a mastery of symbol systems and conditional rules (which, for historical or pragmatic reasons, can be commonly found in robotics/AI implementations). According to the most widely adopted embodied phenomenology theory of skill acquisition (Dreyfus and Dreyfus, 2005), there are five stages of progression from novice to expert, whether in, e.g., riding a bicycle, playing chess, or practicing medicine.

To briefly summarize these five stages, as the theory goes, a *novice* (in any domain) learns by appealing to basic rules that can indeed be expressed symbolically as propositions. Even with these conditional rules, the novice cannot necessarily discern what is relevant in the domain. This changes slightly in the next stage, when the *advanced beginner* continues to follow the rules, but gradually begins to notice what perceptions of the domain are relevant. Upon reaching the third stage, *competence*, the practitioner gains an appreciation of the vastness of domain-relevant nuances, along with the recognition that a list of rules could not be exhaustive; even if such a list could be near comprehensive, it would be too unwieldy to manage in real-time interaction. Nevertheless, to cope with the domain, some rule-like responses remain helpful at this stage. The fourth stage, *proficiency*, finally overcomes the appeal to rule-like responses with an embodied ability to discern relevant situational nuance. However, the proficient practitioner continually reaches decision-making junctures that require a considered evaluation of different pathways forward. In the final stage, when *expertise* is attained, the expert seamlessly selects a pathway forward, rather than interrupting the “flow” (Csikszentmihalyi, 1990) for a considered evaluation. This form of embodied expertise is also described as “absorbed coping,” referring to the phenomenological absorption in the interactive situation.

Without objecting to this characterization of embodied expertise as irreducible to symbols and rules, it is possible to explain the underpinnings of the stage progression using AIF simply by viewing the progression in reverse. If expertise is regarded as having a highly developed generative model of the agent/environment relationships within the domain, then the preferred future realized through active inference is the attainment of the implicit or explicit goal (cycling across the terrain or defeating the chess opponent). Through experience (i.e., empirical prior-based model updating of reliably invariant modality covariances), the agent discovers how to exploit the relevant affordances—the local minima of uncertainty in the generative model state transitions—to achieve the preferred outcome using domain-specific policies.^12^

By working backwards through the progression (moving from expert to novice), it becomes clear that without sufficient experience, the generative model has yet to become sufficiently “attuned” (a Gibsonian term) to the domain; some scaffolding is needed to stabilize the domain-specific interactions. The earlier the stage, the more scaffolding is needed, such that the novice relies almost exclusively on scaffolding (which need not be symbol and rule-based, as it could also be based on mimicry of experts). Any scaffolding presumably also serves to orient the non-expert practitioner to the relevant regularities that facilitate the progression. Note that, when learning to ride a bicycle, training wheels do not directly contribute to learning the cycling skill, but rather, they serve as supportive scaffolding to position the bicycle perpendicular to the ground until the relevant regularities for remaining perpendicular independently have been sufficiently learned.

An interesting robotics application of domain-specific sensorimotor skills is found in the notion of policy reuse and adaptation (Rosman et al., 2016). From an AIF perspective, this parallels an equivalent phenomenon in humans. For example, given the ability to ride a standard bicycle, and confronted with an unfamiliar old-fashioned penny-farthing, an agent could glean from the similar seat, handlebar, wheel, and pedal configuration that the bicycle-riding policy could be reused to ride the penny-farthing, with some necessary adjustments.

A real-world example in which a policy was adapted from a source to a particularly divergent target is the cultural advent of skateboarding, which was based on surfing.^13^ Even though there are extreme differences between surfboard fins and skateboard wheels, ocean and pavement, the early skateboarders recognized the embodied motion similarities between the domains. In this case, a certain cross-domain policy identity is maintained through reuse and adaptation that focuses on the complex spatiotemporal nesting required in both practices involving body, board, and traversal surface: the interactive precision-weighting required for short timescale, rapid adjustments, and the simultaneous progressively longer timescales of extended maneuvering. The Gibsonian concept of “resonance” appears to be appropriately matched to such complex situated activity, in which the agent’s multiscale embodied neurodynamics “resonate” with the multiscale environmental dynamics, following experiential attunement to the relevant regularities (Teques et al., 2017; cf. Raja, 2017).

### 6.3 The Frame Problem

At several points above, we have referred to the agent’s identification of what is relevant or significant in a situation, which appears to run up against the frame problem. To recap, the frame problem holds that given actions that alter limited aspects of a situation, or given relevance-altering shifts in situational context, there is no clear mechanism to appeal to by which irrelevant situational aspects can be easily ignored. Dreyfus (1992) famously proposes that embodiment obviates the frame problem in a way that symbolic AI implementations cannot. He goes further still and proposes that even typical subsymbolic AI cannot overcome the problem; he finds some promise in Freeman’s neurodynamics (Dreyfus, 2007), although his analysis of why this shows promise is limited. Given the convergences between Freeman’s neurodynamics and AIF (Friston, 2008, 2010; De Ridder et al., 2014), it is not surprising that the latter should offer the robust response to the frame problem Dreyfus anticipated.

It is worth briefly restating the nature of neural computation in AIF, due to its substantial difference from the computation of input, symbols, propositional logic, and other common associations. Even the convenient shorthand used by neuroscientists and others that the brain “is” Bayesian or “implements” Bayesian models can lend itself to misunderstanding AIF’s ontological commitments. Essentially, given synaptic connectivity and transmission patterns, it is possible to model them mathematically. It is rarely misunderstood when equations are used to descriptively model a planet’s orbit in order to predict its positions—most people do not assume that this approach suggests the planet itself is computing anything (nor that the planet’s material complexity is “reduced” or “eliminated” in the pragmatic abstraction of a mechanistic orbital model). Analogously, by appeal to the broader theoretical context of AIF, it can be stated that there are transformations in the dynamic neurobiological substrate in the service of the environmentally embedded body that can be mathematically modeled in terms of probability distributions. Thus, embodied and embedded brain activity can be modeled as the computation of these distributions. That the calculations should be Bayes-approximate within AIF results from implicit pragmatic efficiency directives (arising from the constraints laid out in Sections 2 and 3), such as “extrapolate from experience” (empirical priors), “context matters” (hierarchical model architecture), and “when expectations are not met, re-assess” (respond to surprisal through model updating, precision-weighting, or abduction, depending on particulars about the accumulation of prediction error).

The frame problem, in its many incarnations, can be summarized in a single question: How does an agent know what is significant in an interactive situation? AIF answers with its own unique breakdown. The first level of the breakdown is that the agent can be either open or closed to potential significance. This is overlooked by most other accounts, which take openness to significance for granted, thereby missing the ecologically common phenomenon of habits. In AIF, habits can be regarded as context-free responses that are established by their invariance across multiple conditions (FitzGerald et al., 2014). When we act out of habit, we merely “go through the motions,” suppressing any potential significance that might otherwise be contextually relevant.

Apart from habit, when the agent is open to potential significance, AIF points to a second-level breakdown of possible outcomes (when potential significance arises in a situation). Given that the active agent always entertains a repertoire of plausible policies within its generative model, there is a fundamental relationship between policy selection and the expected free energy within the policy or model space. Given that expected free energy scores the epistemic affordance of alternative policies on models, there is an inbuilt imperative to select *significant* or *relevant* actions. Significance in this instance is related to the epistemic, uncertainty-reducing component of expected free energy, while relevance can be construed in relation to prior preferences about ultimate actions. When a potentially significant aspect of the environment recruits a policy, it becomes relevant; this is equivalent to the notion of a “solicitation” in affordance theory and phenomenological philosophy (see Bruineberg and Rietveld, 2014; Bruineberg et al., 2016). In short, the significance or relevance is an integral aspect of FEM by which the frame problem is dissolved.

**Figure 1:**
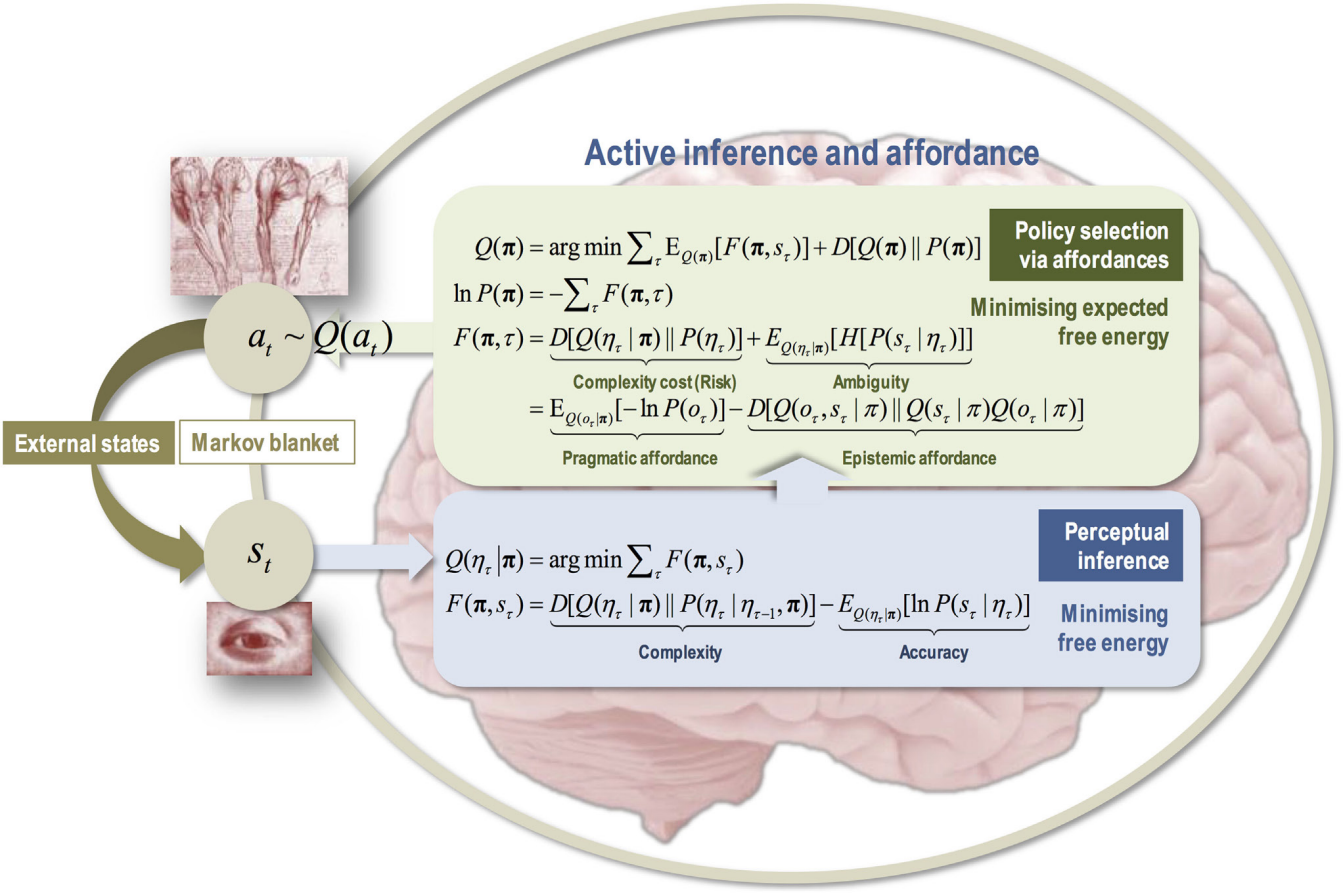
Bayesian mechanics and active inference. This schematic summarizes the formal aspects of active inference in terms of minimizing variational free energy. It describes a generic (active) inference scheme that has been used in a wide variety of applications and simulations; ranging from games in behavioral economics (FitzGerald et al., 2015) and reinforcement learning (Schwartenbeck et al., 2015) through to language (Friston et al., 2017c) and scene construction (Mirza et al., 2016). The details of this scheme are not essential to understand the arguments in the main text: they are presented here for interested readers, with a special focus on how *affordance* emerges from minimizing (expected) free energy, under a generative model of the world. In this setup, discrete actions solicit a sensory outcome (*s*) that informs approximate posterior beliefs about external or hidden states of the world (η). This Bayesian belief updating can be described as minimizing variational free energy *F*(π, *s*) under a set of plausible policies (π). Here, a policy comprises a sequence of actions (*a*). The approximate posterior beliefs are then used to evaluate expected free energy *F*(π, τ) and subsequent beliefs about action; namely the *affordances* that underwrite policy selection. In other words, affordance corresponds to inference about action, where the most likely policy (to be selected) is the policy that minimizes expected free energy in the future. Q(η|π) denotes beliefs about hidden states in the future, given a particular policy and Q(π) are posterior beliefs about the policies currently being pursued. Free energy is just the difference between *complexity* and *accuracy*. In other words, an approximate posterior with a low free energy provides an accurate but simple explanation for sensory input. Expected free energy can be similarly decomposed into expected complexity (i.e., complexity cost or *risk*) and expected inaccuracy (i.e., *ambiguity*). Complexity can be regarded as the divergence (denoted by the Kullback–Leibler divergence *D*) between what one expects to happen under a particular policy and what one would prefer *a priori*. Ambiguity is the loss of a precise or definitive mapping between external states of the world and observed sensory states (as quantified by entropy, denoted by *H*). An alternative decomposition of expected free energy is in terms of *epistemic* and *pragmatic affordance*: see main text. Note a subtle but important aspect of this construction; namely, posterior beliefs about policies are based on their expected free energy, which includes the (path integral) of free energy *per se*. This is interesting from several perspectives. It means that the agent has to infer what it is doing and, implicitly, its own action. In other words, beliefs about action are distinct from the active states of the agent’s Markov blanket (namely the sensory and action states that separate internal from external or hidden states). This means the agent has to predict how it will behave and then verify those predictions based on sensory evidence. This implicit inference means that the agent has to garner evidence for its own behavior. This is the role of the free energy. Namely, free energy *per se* provides evidence that a particular policy is being pursued, while expected free energy scores its prior probability. In summary, agents (will appear to) have beliefs about their behavior—beliefs that endow them with a sense of purpose, in virtue of the prior preferences that constitute risk. In effect, this enables agents to shape their sensorium. Please see Friston et al. (2017b) for technical details and Friston et al. (2017a) for a discussion of how this belief updating might be implemented in a brain.

This argument rests upon appreciating that expected free energy can be decomposed into two parts (Figure 1). Variational free energy *per se* can always be decomposed into accuracy and complexity terms. This appeals to the Bayesian interpretation of variational free energy as an approximation to (or lower bound on) Bayesian model evidence. On this view, Bayesian model evidence is effectively *simplicity* plus *accuracy*.^14^ But what about *expected* free energy? It transpires that *expected accuracy* is the expected probability of obtaining preferred outcomes, while *expected simplicity* is epistemic affordance, namely, the resolution of uncertainty or information gain afforded by the outcomes anticipated under any particular policy. This intrinsic value of a particular policy or model appears in many guises, most notably as intrinsic motivation in robotics (Oudeyer and Kaplan, 2007; Schmidhuber, 2010), the value of information in economics (Howard, 1966), and Bayesian surprise in models of exploration and visual searches (Schmidhuber, 1991; Itti and Baldi, 2009).

Ultimately, without the input/output model, the core difficulties associated with the frame problem—when to sample input, what to sample as input, what to do with input, or what becomes of fixed output—do not arise. There is only the generative model’s accommodation of sensory perturbations in terms of hidden causes. By incorporating epistemic imperatives into the (Bayesian model) selection of policies in AIF, the broad frame problem never manifests. This is because novel information is not pre-screened for relevance, but instead is rendered relevant or significant when it leads to model updating or the selection of a new policy, and irrelevant or insignificant when it does neither. Note that the latter case holds irrespective of benefit or cost, given that the non-assimilation of novel information may be helpful (e.g., metabolic savings) or harmful (e.g., missed opportunity).

This approach also avoids concerns about the inadequacy of fixed representational encoding accounts of perception (Bickhard, 2008), given that in AIF, environmental information can serve multiple context-dependent relational roles in situated interaction (cf. Pylyshyn, 1999). Moreover, the logical frame problem is obviated by the probability distributions of the generative model—the agent interacts with the environment on the basis of expected model extrapolations, so continuous sensory sampling is unproblematic: samples either confirm expectations or produce surprisal (Mirza et al., 2016).

## 7 Self-Reflective Epistemic Foraging: An Opening for Consciousness?

The reservoir of information present with respect to the self and the environment is inexhaustible. Only a small fraction is ever immediately relevant as adaptive behavioral control information. Thus, there are always new sources of potential relevance, as there are many possible signals in the noise (Dennett, 1991). While many discussions of AIF center on epistemic foraging in the environment, it is also possible to consider epistemic foraging of the self, also a rich source of signals in the noise (Seth, 2013; Seth and Friston, 2016).

Thus far, we have primarily addressed control information, noting that it can also be gleaned from conscious phenomenal sensation (Seth et al., 2012). Enhancing the generative model through exploration, also known as epistemic foraging, provides potential future control information. However, when new significance arises, it is not necessarily immediately subsumed as control information. Consider hearing a fellow diner’s request to “pass the salt.” Given situated language learning (Diessel, 2006), words provide evidence for the most apt generative model or policy (Lupyan and Clark, 2015), enhancing the control information for the relevant modification of the thermodynamic substrate (identifying, grabbing, and passing a nearby salt shaker). Nevertheless, the request is also appreciable as a phenomenal sensation that can be further epistemically foraged. For instance, the diner’s shaky tone of voice might indicate an emotional state that was not immediately relevant to passing the salt, but may become relevant in social interaction, leading to an enquiry about their wellbeing (Filippi et al., 2017).

What should facilitate such inquiring? When time pressure is low, it is possible to reflectively evaluate information beyond its role in facilitating immediate adaptive behavior. AIF can describe this as the momentary decoupling of aspects of the model from the environment for self-reflective epistemic foraging, while potentially remaining partially environmentally engaged (e.g., thinking about the office during the commute). Having this ability would confer adaptive advantages, such as navigating complex social meaning, as well as more protracted forms of elaborate problem-solving (mentally revisiting a problem from different angles). This example also speaks to the trade-off between epistemic (expected simplicity) and pragmatic (expected accuracy) imperatives that underlie FEM in policy selection. In brief, the trade-off—not dissimilar to an exploration/exploitation trade-off—rests upon the precision of prior preferences. Generally, in a new situation, epistemic affordance would normally dominate policy selection until there is a comfortable familiarity with the lived context; prior preferences can then come into play. Crucially, these prior preferences are themselves inferred in deep (hierarchical) generative models.

A strong candidate for facilitating such self-reflection is also the most apparent correlate of self-consciousness: a mental buffer that underpins introspective awareness. This buffer can be regarded as the substrate of conscious mental simulation, imagination, and internal monolog. The latter would allow for forms of self-reflection, as well as the self-referential fine-tuning of adaptive behavior (“I must remain focused on the road!”). It is relatively uncontroversial to view simulation as contributing to adaptive behavior through mental rehearsal, and imagination as contributing to generating counterfactuals and exposing new affordances, while also enabling the suppression of conscious environmental coupling.

Whatever its genesis and other roles, consciousness appears to be crucial for epistemic foraging in the limitless source of signals in the noise of the self, in a manner wholly consistent with the information-bound AIF elaborated above. Note that bringing consciousness to the table presupposes a generative model of the future that necessarily entails a degree of selfhood and agency. This characteristic of generative models has been referred to as counterfactual richness or depth (Seth, 2015) to emphasize the deep and fictive nature of how (some) agents predict their world and behavior.

Moreover, from the AIF perspective, we can identify a feature that appears to be rare in the animal realm that could be plausibly robotically implemented. Our fundamentally thermodynamically constrained social origins imply a capacity for ethical considerations, at least concerning basic aspects of resource sharing (Cosmides et al., 2010). In this context, consciousness as a buffer for self-reflective epistemic foraging would underpin our ability to evaluate preferred outcomes and inferred policies from a space of possible state transitions—in other words, to evaluate ends and means to ends—on the basis of ethical considerations.

Through conscious, self-reflective epistemic foraging, a self-conscious agent can turn active inference inward, by nuancing model or policy selection to alter its current outcome preference. Also, when a preferred outcome has been selected, an agent can determine whether it ought to infer a policy alternative to the immediate, intuitively inferred policy it would have selected under time pressure.^15^ (This can be thought of as the agent’s self-referential policy to realize a preferred future in which *other* possible ends and means have been duly considered.) With the luxury of time, consciously aware self-reflective agents can individually and cooperatively aim for a deeply considered preferred future, to be reached *via* a deeply considered pathway.

The above speculations are indicative of the manner in which AIF can plausibly connect an agent’s consciousness to its embedding in progressively larger social organizations. The mechanistic—yet radically non-reductive—explanatory underpinning of this embodied, embedded account of individuals and society inherently includes their openness to vast cultural proliferations and indeterminate futures.

## 8 Conclusion: At the Crossroads of Natural and Artificial Embodied Cognition

We have seen above why, in contrast to common assumptions, AIF *opposes* the mechanistic to the reductive. If AIF were applied to developing a humanoid robot that would approximate a human being, it is clear that its embodied apparatus must be more than just for show. The mechanical actuation would need to furnish the proprioceptive sensing aspect of the generative model that would exhibit reliably invariant covariance with exteroceptive sensing. For this extero- and proprioceptive coupling to be biomimetic, the sensing should have the same constraints as our biologically inherited apparatus, such as a limited visual range that is extended by bodily movement. Assuming a neuromorphic information integration apparatus were also implemented, we could expect robotic interoception to identify environmentally relevant quantities such as energy requirements (“hunger”) and bodily damage (“pain”).

So far, none of this would require consciousness, though it could achieve basic adaptive behavior. For a more deeply situated robot, we would need to add a minimal mechanism for distinguishing foreground from background, and one for differentiating between quasi-conspecifics (others of the same make or possibly humans as well). This could serve to fulfill the requirement of social grounding that would in principle pave the way for cooperative communication strategies, such as gesture and language.

With an appropriate buffer of interoceptive self-awareness, the robot could epistemically forage within this buffer for additional relevant signals than those it first identifies in the environment. Through the usual human routes of upbringing and education, it could also be taught to evaluate the consequences of its actions, to weigh preferred ends and available means by considering their potential impact on itself and others. The process of learning to appreciate counterfactual outcomes would be enhanced by a capacity for valenced esthetic experiences (“emotions”). This suggests a broadly socially situated (humanlike) role for emotional regulation (see, e.g., Sell et al., 2017), which differs considerably from current robotic implementations of pseudo-emotional states (e.g., Moshkina et al., 2011).

It would be within reason to describe the set of processes in AIF as algorithms, which raises the question: what implications does this have for our understanding of humans? There have been many recent discussions of algorithmic bias in computer systems said to reflect the bias of the human system designers. This is not surprising, given any disembodied algorithm based on a reductive input/output model. With AIF, however, we can make sense of natural and artificial ecologically and socially situated embodied agents. Agents with this specification would interactively probe and learn the apparent regularities of their world. At the same time, with sufficient complexity, they would have the capacity to critically evaluate their own generalizations from past environmental exposure, to identify when forms of bias are detrimental, and to engage in meaningfully value-laden self-corrective recalibration (while of course this provides no guarantees, even for humans; see, e.g., Bang and Frith, 2017; Holroyd et al., 2017).

To summarize: by appeal to the principle of FEM, we can descriptively account for a long view that takes us from elementary particles to embodied biological agents. In an ecological context, the emergence and behavior of these agents—underpinned by a cybernetic relationship between thermodynamics and information—can be understood to plausibly facilitate the evolutionary development of life. On a long enough time scale, under contingent circumstances, FEM is sufficient to yield the coevolutionary development of mutually adaptive, highly complex agents and niches, as we see in human culture, especially in our pragmatic and epistemic foraging behavior, which fundamentally includes socially cooperative and self-reflective capacities. Taking all of this into account, AIF suggests a possible approach to the biomimetic modeling of human agents that in principle would exhibit humanlike embodied cognition. Such agents would plausibly be conscious in most senses of the word.

## Author Contributions

AL, AC, SR, and KF made substantial contributions to the conception and/or design of the work, its drafting and/or critical revision, and approved for publication its content.

## Conflict of Interest Statement

The authors declare that the research was conducted in the absence of any commercial or financial relationships that could be construed as a potential conflict of interest.

## Footnotes

^1^For a related approach in philosophy of science, see, e.g., Bechtel (2014) and Bechtel and Abrahamsen (2007).

^2^This, of course, could be millions of years.

^3^We specify “typically” here as a nod to the Fluctuation Theorem (that generalizes the second law to non-equilibrium systems). In brief, the Fluctuation Theorem says that the probability of entropy decreasing vanishes as the observation time or size of the system increases (Evans and Searles, 2002). In other words, at a microscopic level, it is possible to have transient decreases in entropy, but the probability of this occurrence quickly becomes almost zero, over time.

^4^Maxwell’s demon is a thought experiment proposed by James Clerk Maxwell to account for violations of the Second Law of Thermodynamics (Maxwell, 1871, pp. 308ff.). Subsequently, it was realized that even Maxwell’s demon complies with the Second Law in virtue of Landauer’s principle, namely, that “any logically irreversible manipulation of information, such as the erasure of a bit or the merging of two computation paths, must be accompanied by a corresponding entropy increase in non-information-bearing degrees of freedom of the information-processing apparatus or its environment” (Bennett, 2003).

^5^We will use the term of variational free energy (in information theory and Bayesian statistics) to distinguish it from thermodynamic free energy in FEM.

^6^Note that this is an instance of Landauer’s principle described in Footnote 4, speaking to the fact that there is no free lunch when it comes to trading information for energy—in any process, the two are essentially the same.

^7^Note that we are again appealing to the good regulator theorem. In other words, there is a homology between the complexity of the world being regulated and the good regulator that must embody a model of that world.

^8^See Yahiro et al. (2017) for preliminary empirical support of this premise; their experimental findings point to different physiological pathways, e.g., low environmental temperature leading to involuntary shivering vs. the phenomenal sensation of coldness leading to voluntary warmth-seeking behavior. On the complex interplay between phenomenal sensation and preconscious information, see Sergent et al. (2013).

^9^For discussion, see Sprevak (2005).

^10^Our description of active inference here will be based largely upon discrete time and state space generative models (e.g., Markov decision processes). These are simpler to handle in terms of their numerics (and possibly conceptually); however, the same principles apply to the continuous state space models usually considered in Bayesian filtering and predictive coding formulations of active inference.

^11^See Albrecht et al. (2016) for an implemented reinforcement-learning-based decision-making model defined in terms of such probabilities (expectations).

^12^In performing arts such as music, skilful policies may relate to actualized or simulated coordination in improvisation, performance, and compositional practices (see Linson, Forthcoming).

^13^The early skateboarders were “replicating on dry land the surfer’s traverse across ocean surface and close sensing of changing wave forms. Through surf-related moves, skaters recombined body, board and terrain, simultaneously copying one activity (surfing) while initiating a second (skateboarding)” (Borden, 2001, pp. 31–33).

^14^Note that minimizing variational free energy implicitly minimizes complexity and associated computational costs—*via* Landauer’s principle—that link thermodynamic free energy to variational free energy. In other words, the path of least variational free energy is, thermodynamically, Hamilton’s path of least action.

^15^Time pressure is accommodated in active inference by appealing to Hamilton’s principle of least action. In other words, it is the expected free energy over time that counts, where unexpected energy corresponds to an action. Put simply, for adaptive efficacy, it is much better to reduce free energy quickly, to an imperfect level, than to spend lots of time reducing it to its minimum.

## Appendix

### Glossary of Terms

In Bayesian statistics and machine learning, several common terms have technical meanings. This glossary defines the way in which we use key terms in the current article.

*Free-energy*: an information theory measure that bounds (is greater than) the surprise on sampling some data, given a generative model.

*Entropy*: the average surprise of outcomes sampled from a probability distribution or density. A density with low entropy means, on average, the outcome is relatively predictable. High entropy denotes unpredictability and uncertainty.

*Surprise, surprisal*, or *self-information*: the negative log-probability of an outcome. An improbable outcome is, therefore, surprising. Negative surprise is the same as *log evidence*; namely, the logarithm of Bayesian model evidence.

*Bayesian surprise*: a measure of salience based on the divergence between the posterior and prior probability densities. It measures the information gain obtained by updating the priors to posteriors.

*[Kullback–Leibler] Divergence*: information divergence, information gain, or relative entropy. The divergence is a (non-commutative) measure of the difference between two probability distributions.

*Generative model*: a probabilistic model that generates consequences (i.e., data) from their causes (i.e., model parameters). A generative model is also known as a forward model and is usually specified in terms of the likelihood of getting some data given their causes (parameters of a model) and priors on the parameters.

*Prior*: the probability distribution or density over the causes of data that encode beliefs about those causes prior to observing the data.

*Empirical prior*: priors that are induced by hierarchical models; they provide constraints on the recognition density is the usual way but depend on the data.

*Conditional density* or *posterior density*: the probability distribution over causes or model parameters, given some data; i.e., a probabilistic mapping from observed consequences to causes. In Bayesian inference, the prior is updated—on the basis of observations—to become a posterior, according to Bayes rule.

*Model evidence*: in Bayesian statistics, the model evidence is the probability that observed data were generated by a particular generative model. The negative logarithm of model evidence is surprise or self-information in information theory.
